# Loss of Olfactory Function—Early Indicator for Covid-19, Other Viral Infections and Neurodegenerative Disorders

**DOI:** 10.3389/fneur.2020.569333

**Published:** 2020-10-26

**Authors:** Heike Rebholz, Ralf J. Braun, Dennis Ladage, Wolfgang Knoll, Christoph Kleber, Achim W. Hassel

**Affiliations:** ^1^Center of Neurodegeneration, Faculty of Medicine/Dental Medicine, Danube Private University, Krems, Austria; ^2^Institut de Psychiatrie et Neurosciences de Paris (IPNP), UMR S1266, INSERM, Université de Paris, Paris, France; ^3^GHU Psychiatrie et Neurosciences, Paris, France; ^4^Center of Chemistry and Physics of Materials, Faculty of Medicine/Dental Medicine, Danube Private University, Krems, Austria; ^5^Universitaetsklinikum Köln, Cologne, Germany; ^6^Austrian Institute of Technology, Vienna, Austria; ^7^Institute of Chemical Technology of Inorganic Materials, Johannes Kepler University Linz, Linz, Austria

**Keywords:** COVID-19, anosmia, hyposmia, SARS–CoV-2, normosmia

## Abstract

The loss of the senses of smell (anosmia) and taste (ageusia) are rather common disorders, affecting up to 20% of the adult population. Yet, this condition has not received the attention it deserves, most probably because per se such a disorder is not life threatening. However, loss of olfactory function significantly reduces the quality of life of the affected patients, leading to dislike in food and insufficient, exaggerated or unbalanced food intake, unintentional exposure to toxins such as household gas, social isolation, depression, and an overall insecurity. Not only is olfactory dysfunction rather prevalent in the healthy population, it is, in many instances, also a correlate or an early indicator of a panoply of diseases. Importantly, olfactory dysfunction is linked to the two most prominent neurodegenerative disorders, Parkinson's disease and Alzheimer's disease. Anosmia and hyposmia (reduced sense of smell) affect a majority of patients years before the onset of cognitive or motor symptoms, establishing olfactory dysfunction as early biomarker that can enable earlier diagnosis and preventative treatments. In the current health crisis caused by SARS-CoV2, anosmia and dysgeusia as early-onset symptoms in virus-positive patients may prove to be highly relevant and crucial for pre-symptomatic Covid-19 detection from a public health perspective, preceding by days the more classical respiratory tract symptoms such as cough, tightness of the chest or fever. Thus, the olfactory system seems to be at the frontline of pathologic assault, be it through pathogens or insults that can lead to or at least associate with neurodegeneration. The aim of this review is to assemble current knowledge from different medical fields that all share a common denominator, olfactory/gustatory dysfunction, and to distill overarching etiologies and disease progression mechanisms.

## Introduction

The current Covid-19 pandemic is reaching more and more regions, countries and tens of thousands of new patients daily. One symptom of the disease has not been fully recognized until the end of April at which point, the reduction or the loss of olfactory function caused by the SARS-CoV2 virus was added to the list of Covid-19 symptoms by the WHO, in addition to the well-defined symptoms such as fever, cough and shortness of breath (https://www.who.int/health-topics/coronavirus#tab=tab_3).

Since other coronaviruses have been shown to be neuroinvasive, the question arises if SARS-CoV2 uses the neuroepithelium as a port of entry to the brain, causing olfactory dysfunction through action on the peripheral or the central components of the olfactory system. According to the so-called vector hypothesis, it is also possible that this virus will cause neuronal damage in other non-olfaction-related parts of the brain, a hypothesis that needs to be thoroughly addressed in the future.

This review assembles relevant studies on changes in the senses of smell and taste as indicators of peripheral and central pathologies induced by various pathogens or environmental toxins.

The loss of normal olfactory function is categorized according to whether a loss is complete (anosmia) or partial (hyposmia), compared to the norm (normosmia). Often co-affected is the sense of taste which, when lost, is termed ageusia and, when disturbed, dysgeusia. The majority of persistent anosmia or hyposmia is caused by upper respiratory tract infections, head injury or nasal sinus pathology (1). In rarer cases, exposure to environmental chemicals (2), medical interventions such as radiation or chemo-therapy (3, 4), surgical procedures in the nasal areas such as septoplasty, rhinoplasty, can be at the origin of olfactory dysfunction (5, 6). Finally, medical conditions such as intranasal growths, epilepsy, psychiatric disorders, hypothyroidism, renal and liver disease can cause anosmia/hyposmia (7). It is important to distinguish between a preexisting disability and an acquired loss of the sense of smell, as some substances (e.g., products of asparagusic acid**)** may not be smelled or tasted due to a genetic condition (8).

At first glance, the loss of smell may not be life-threatening, while in fact there is a statistical association between olfactory acuity and mortality (9). Impaired olfaction negatively affects quality of life, enjoyment of food, creates difficulties in maintaining personal hygiene, leads to greater incidence of depression and isolation, and affects overall physical and mental well-being (9–12). Loss of olfactory function also impairs the ability to detect dangerous smells, such as fire, environmental toxins, leaking natural gas, and spoiled food and therefore, indirectly, is life-threatening (9, 10).

## The Physiology of Senses of Smell and Taste

**Figure 1:**
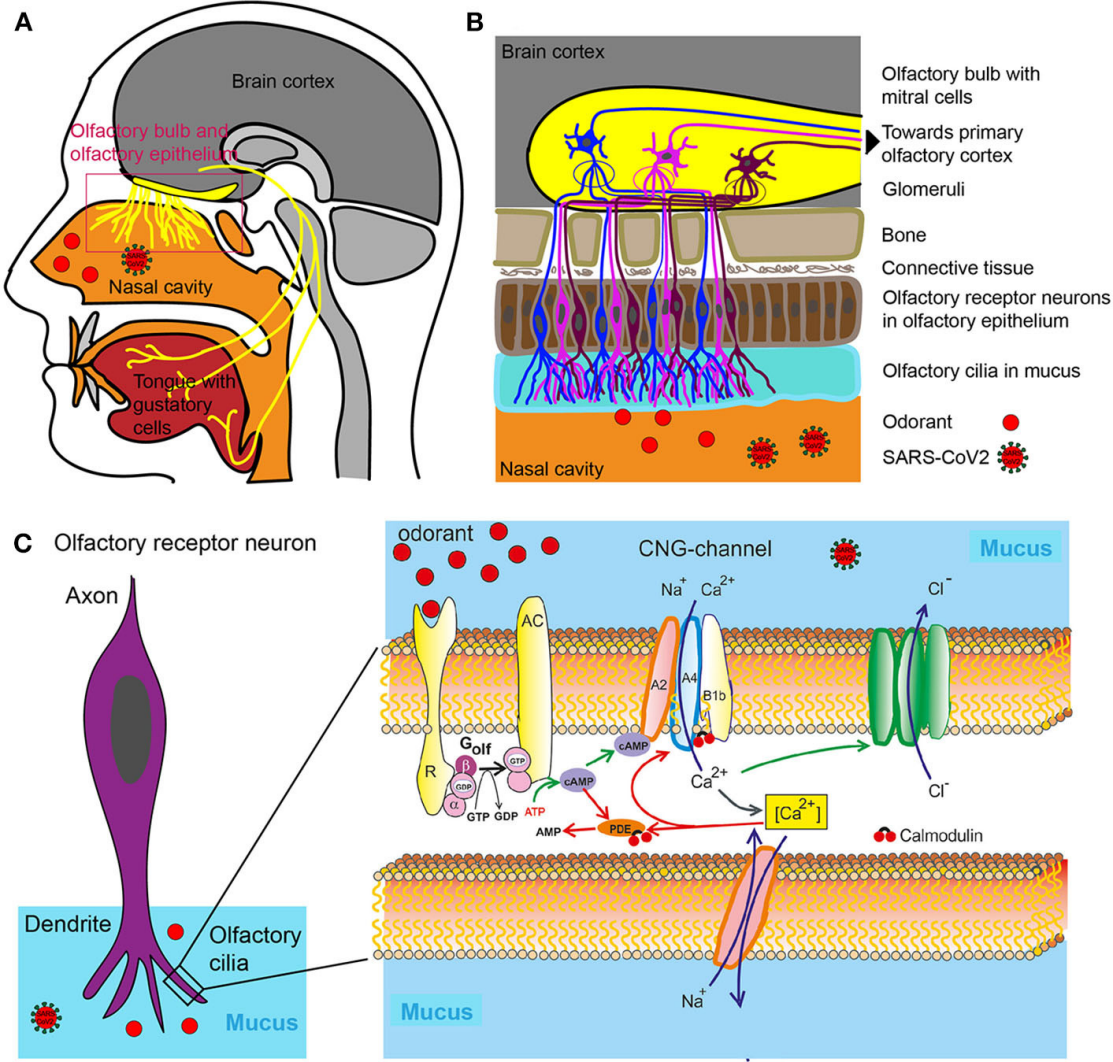
Olfactory system. **(A)** Head sagittal section showing the olfactory and gustatory systems. **(B)** Olfactory bulb, olfactory epithelium with olfactory receptor neurons. **(C)** Left: Olfactory receptor neuron with olfactory cilia. Olfactory receptor neurons are bipolar neurons with a dendrite carrying a crust of sensory cilia. Right: Part of an isolated olfactory cilium illustrating processes upon odorant binding. The green arrows show activating, the red adapting processes. AC, Type III adenylate cyclase; AMP, adenosine monophosphate; cAMP, cyclic adenosine monophosphate; [Ca^2+^], intracellular Ca^2+^ concentration; CNG, cyclic nucleotide-gated ion channel; Golf, olfactory G protein; PDE, phosphodiesterase; R, odorant receptor.

Smelling requires the intricate interaction between the nasal cavity which receives an odorant stimulus and its transmission via a series of interconnected neurons and brain structures which then compute various concomitant stimuli into the notion of a specific smell (Figure 1).

An odor is either a single molecule (e.g., hydrogen sulfide that smells of rotten eggs) or is composed of a combination of molecules termed odorants with specific chemical and structural properties which are recognized and bound with varying degrees of affinity by the so-called odorant receptors located in the nasal cavity (13) (Figures 1A,B).

The mammalian odorant receptor (OR) gene family comprises more than 1,000 members, which represents the largest G-protein coupled receptor (GPCR) gene family in the mammalian genomes (14). The human OR gene family encompasses 857 members (15) (https://genome.weizmann.ac.il/horde/). Thereof up to 391 encode functional olfactory receptors (ORs), whereas 466 OR gene family members are pseudogenes, i.e., non-functional sections of DNA (15). The Ca^2+^ influx in turn opens a Ca^2+^-activated Cl^−^ channel leading to efflux of Cl^−^, further depolarizing the cell and triggering an action potential (Figure 1C). Olfactory cells usually react only briefly to stimulation with odorants. Even if odorant molecules are continuously offered, the cells only react for a few seconds, then they become silent—they adapt. The adaptation itself is inhibited by various mediating processes that terminate the receptor flow. These processes are controlled by the Ca^2+^ ions which enter by cyclic nucleotide-gated ion channel (CNG**)** into the cilia. So this is a Ca^2+^-mediated feedback inhibition. The olfactory cells' CNG channels are continuously Calmodulin bound. If Ca^2+^ enters the cell, it binds to calmodulin and causes a change in conformation. This, in turn, leads to a closing of CNG channels: The signaling cascade is therefore interrupted. Calmodulin also mediates other adaptive mechanisms. The enzyme phosphodiesterase (PDE) is produced by Ca2^+^ /Calmodulin activation. Phosphodiesterase splits cAMP and reduces second messenger concentration (Figure 1C) (16).

The reason for the large number of different odor receptors is to provide a system for discriminating between as many different odors as possible (17). Odorants themselves are volatile substances, members of different chemical classes (e.g., alcohols, aldehydes, ketones, carboxylic acids, esters, aromatic, and sulfur-containing compounds). Upon binding and being activated by the specific odorants, all neurons expressing the same odorant receptor convene in deeper structures in the nasal cavity termed the glomeruli (18) (Figure 1B). Because several receptor types are activated due to different chemical features of the odorant, several glomeruli are activated. The combination of glomeruli activation encodes the different chemical features of the odorant. From the glomeruli the stimulus is relayed to the olfactory bulb where olfactory neurons synapse with Mitral cells and from where the sensory information is relayed to parts of the brain such as olfactory cortex and other areas (Figure 1B). The brain then puts the pieces of the activation pattern back together to identify and perceive the odorant. For a review on the physiology of smell see (19).

The senses of smell and taste are intrinsically linked. Flavor perception is an aggregation of taste and smell sensory information. During the process of mastication, the food mash releases odorants into the nasal cavity, which are registered through the odorant receptors. The tongue via specific receptors on taste cells that are bundled together to form taste buds can distinguish only among five distinct qualities of taste (sweet, salty, sour, bitter and umami), while the nose can distinguish among literally hundreds of thousands of odors (17).

The nose, or more precisely dendrites of olfactory neurons located in the olfactory epithelium, is a structure that exposes the brain to the outside world without the protection of the blood brain barrier (20). Olfactory neurons (the neurons expressing odorant receptors) project directly to the olfactory bulb, which is a component of the central nervous system (CNS) without an intervening synapse (Figure 1B). This special feature is exploited in the development of intra-nasal delivery tools to introduce therapeutic molecules that would otherwise not pass through the blood-brain barrier (BBB). Pathogens are using the same route to penetrate higher brain regions through olfactory neurons and the bulb. It has long been known that pathological infections of the brain can be caused via entry through the nasal mucosa. In one extreme measure this knowledge was applied to prevent infection: In Canada, in the 1930ies, the olfactory epithelia of school children was cauterized to prevent the spread of the polio virus (21). More recently, the infectious prion protein was found in various central parts of the olfactory system including the primary olfactory cortices of patients with Creutzfeldt–Jakob disease. And, reminiscently to the ongoing early SARS-Cov2 studies, patients with Creutzfeldt–Jakob disease first observed anosmia and changes in taste and smell as symptoms (21, 22).

The taste system is more resilient to injury than the olfactory system (23). The reason for this is that multiple nerves transmit taste information to the brain: the facial nerve, the glossopharyngeal nerve and the Vagus nerve (Figure 1A). They all supply gustatory information and help to protect an individual from a generalized loss of taste as a result of an isolated peripheral nerve injury (23). Furthermore, the trigeminal system contributes to taste by sensing qualities such as spicy hot, tingling, burning, and cooling (24).

## Anosmia Caused by SARS-CoV2

Already before the Covid-19 pandemic, human coronaviruses such as CoV 229E were known to cause olfactory dysfunction (25), however the scale and urgency of Covid-19 pandemic has precipitated our knowledge on the effect of coronaviruses on olfaction. Following an accumulation of publications in the recent weeks, it has become widely accepted that anosmia or hyposmia is induced by SARS-CoV2. In fact, several studies have demonstrated that hyposmia or anosmia as well as ageusia are common symptoms (26–30). In a survey, smell and taste loss were reported in 68 and 71% of subjects, respectively, compared to 16 and 17% in healthy controls. This study also reported that 74% of patients experienced a resolution of anosmia at recovery (31). Interestingly, the patient cohort with anosmia also was the cohort that was affected by Covid-19 to a lesser degree of severity it was not the group that needed hospitalization. Thus, conversely, admission for Covid-19 was associated with intact sense of smell and taste, increased age, diabetes, as well as respiratory failure (31–33).

In another survey, 39% of Covid-19 cases reported smell and taste dysfunction, compared to 12.5% of controls. Again, anosmia, and more so ageusia, affected significantly more of the younger patients (34). This was also the case in another survey where 47% of Covid-19 patients reported anosmia, while 85% reported dysgeusia. These symptoms abated after on average 9 days, however in contradiction to other studies, the authors report that the olfactory loss started days after the known onset of infection (35). The inverse correlation of disease severity and penetrance of smell symptoms in not understood, however one plausible hypothesis is a differential immune response within the nasal structures: A higher immune response is expected to weaken the sense of smell but may prevent the virus from spreading to deeper respiratory organs such as the lungs, while, inversely, a lesser and localized immune response may allow viral propagation and spreading to the lower respiratory tract with its known life-threatening complications (26, 36).

Up to date, in addition to several questionnaire-based surveys, only one study was published where olfaction was assessed with a validated smell test. In an Iranian cohort, the University of Pennsylvania Smell Identification Test (UPSIT), a 40-odorant test, showed that 98% of Covid-19 patients exhibited smell dysfunction and 25% were fully anosmic, whereas age and sex matched controls did not exhibit these deficiencies. Deficits were evident for all test odors. In this study, few patients presented with taste loss, which, as the authors speculate, may in fact be due to lack of ability to smell rather than taste. However, this question was not further investigated in this study, the authors argued that in their cohort taste loss always coincided with smell loss and that taste is constituted, in addition to taste bud activation, by volatile stimuli (smells) that enter the mouth from the nasopharynx during deglutition. The difference to the earlier mentioned studies where ageusia was more prevalent than hyposmia/anosmia could be due to objective smell taste testing in this study as opposed to the questionnaires used in the other studies.

It has been shown that subjective assessment of changes in olfactory function do not align with measured changes determined with standardized smell tests such as sniffing sticks. Self-reporting consistently showed that individuals underestimate the extent of their hyposmia (37, 38). In a large USA-wide study, 12.4% of older adults reported their sense of smell as fair or poor (using a 5-point Likert scale), whereas 22.0% had objective olfactory dysfunction. Among those with measured olfactory dysfunction, 74.2% did not recognize it (38).

Like its closest relative SARS-CoV1, SARS-CoV2 binds through its receptor-binding domain (RBD) to human angiotensin converting enzyme ACE2 receptor protein, however SARS-CoV2 binds with higher affinity than SARS-CoV1 (39). ACE2 is ubiquitously expressed in human organs including lung parenchyma, renal and urinary tract, human airway epithelia, lymphoid tissues, reproductive organs, vascular endothelium, and brain while the nasal mucosa or the gastrointestinal tract exhibit particularly high expression levels and may therefore be more vulnerable to viral onslaught (40). There is still debate as to which cells in the nasopharynx express Ace2 (41). Interestingly, olfactory receptor cells do not express ACE2, as well as another gene involved in SARS-CoV2 entry (TMPRSS2) implying that damage to the olfactory receptor cells may be mediated through other cells (42). However, sustenuclar cells which support olfactory neurons, express Ace2 as well as TMPRSS2 and are infected by SARS-CoV-2. They may therefore represent the viral entry point to the nasopharynx (43). These findings suggest that SARS-CoV-2 infection of non-neuronal cell types, in particular of sustenuclar cells, leads to anosmia and related disturbances in odor perception in COVID-19 patients (44). Infection of these cells may cause rapid disruption of the epithelium and combined with a possible inflammatory response lead to smell loss (41).

Some upper respiratory tract viruses such as corona or influenza virus have also been shown to target the central nervous system and to lead to neurological symptoms such as encephalopathies, encephalitis, epilepsies and seizures (45). In analogy, in some Covid-19 patients with anosmia/hyposmia, altered mental state and encephalopathy were reported which could attest to the neuroinvasive potential of the virus (46). Thus, anosmia could also be seen as an indicator of wider neurological damage.

The entry to the brain can happen along several pathways:

1. Viruses pass from the nose directly into the brain by entering through peripheral nerve terminals, moving anterogradely and passing through synapses using the machinery of active transport within those cells into the CNS (47–49)
2. Viruses pass from the nose directly to the cerebrospinal fluid (CSF) since the nose is connected to the CSF (50). Whether SARS-CoV2 reaches the central parts of the human brain, has not yet been established, but other coronaviruses (and SARS-CoV1) have been detected in CSF in humans (40) and in the brain (51–54). Studies in which mice where trans-nasally infected with SARS-CoV2 and MERS-CoV showed that these viruses reach the brain via olfactory nerves, the thalamus and brain stem, the area which was most affected (55).
3. Coronaviruses could also use the retrograde neuronal transport through the vagal nerve afferents from the lungs into the CNS or enter the CNS via the gastrointestinal tract within the brain-gut axis of which the vagus nerve is a major component (46).
4. One additional pathway through which human coronavirus may reach the CNS is by passing the epithelium and reaching the blood stream or the lymph (56). Viruses can further infect different myeloid cells to manipulate the innate immunity and to disseminate to other tissues, including the CNS (56, 57).

For SARS-CoV2, these questions of entry and penetration routes will need to be addressed in the future.

Due to the co-expression of ACE2 and nicotinic acetylcholine receptor (nAChR) in many cells, there exists a functional link between them. In smokers the activation of nicotinic acetylcholine receptors leads to an upregulation in the expression ACE2. It has been speculated but not studied that smokers might be at higher risk for SARS-CoV2 infections (58). In reality, the numbers seem to suggest something different. Smokers are less represented in the disease cohort than in the control cohort (42) and, interestingly, seem to be protected from olfactory loss in Parkinson's disease (as discussed in section Anosmia in Aging and Age-Related Neurodegenerative Disease) (59).

Most Covid-19 patients do not suffer from nasal obstruction and reduced air flow as caused by the swelling of the mucosa. One study reports that only 4% of patients with reported olfactory function loss present with additional nasal obstruction (34). This indicates that olfactory loss is caused not by rhinitis (irritation/inflammation of the mucous membrane, nasal obstruction and discharge) but by damage to either the peripheral and/or central components of the olfactory system.

Reports have demonstrated that the use of corticosteroids may escalate Covid-19 infection, while others supported the use in hospitalized patients (60). Clinical trials to address this question are ongoing (61).

## Anosmia Caused by Influenza or Common Cold

Acute viral upper respiratory tract infections are the most common cause of chronic olfactory dysfunction and are responsible for between 11 and 40% of olfactory disorders (62, 63). The causative pathogens are usually viruses leading to common cold or influenza that manifest themselves without rhino-sinusitis. Approximately 20% of common cold cases can be accounted for by a type of coronavirus (non-SARS) and up 30% by a type of rhinovirus. Other viruses are adenovirus, respiratory syncytial virus (RSV) or human parainfluenza virus (64, 65). Generating vaccines against them has proven unsuccessful due their vast genetic variability (66, 67). In 20–30% of cases, the virus that causes it is unidentified.

The influenza virus causes a more serious disease, infects the lungs and is able to cause pneumonia, respiratory failure and death. In the 2018–2019 influenza season, 34,200 deaths were recorded in the USA by the Center for Disease Control and Prevention (https://www.cdc.gov/flu/about/burden/2018-2019.html). Vaccination against Influenza was shown to negatively correlate with the incidence of smell loss, however this study needs confirmation with a larger cohort number (68).

Any of the above-mentioned viruses can lead to post-viral olfactory dysfunction (PVOD), but factors that determine susceptibility are not elucidated. Women are more often affected than men and PVOD usually occur after the 4th decade of life. The onset of olfactory dysfunction as a result of viral infection is usually rapid, and the probability of normalization, often after years of onset, is relatively high, 32–66% of patients eventually recover (10).

A virus can affect the sense of smell by means of two mechanisms of action. The first one, which is seen in other coronavirus infections or in the common cold, but not in SARS-CoV2, is a physical effect: Through local inflammation, the lining of the nasal passages becomes swollen, the mucosa is lined by a film of nasal discharge, thereby hindering the odorant molecules to reach the corresponding receptors and bind to them, resulting in reduced ability to smell. Furthermore, because of such airway obstruction, the airflow is significantly reduced and odorant molecules will enter the cavity in insufficient quantities. In a study by Åkerlund et al. post-viral olfactory disorder developed after healthy human subjects with no previous olfactory deficiencies were inoculated with coronavirus (229E) (69) which is known to induce the common cold. Olfactory testing 4 days after inoculation show a reduction in olfactory function in these patients who developed a cold. The outcome correlated with increased nasal obstruction as measured by airflow and assessment of nasal charge. Thus, in this particular study, olfactory dysfunction during acute phase of infection, not post-virally, is measured and thus may result from both swelling of the nasal mucosa/airflow obstruction and virally induced damage to the olfactory epithelium (69).

Another mechanism involves the virus damaging the olfactory epithelium and peripheral nerves and cells located therein. The exact location of the damage in post-upper respiratory tract infections is not yet known, and it is disputed if the olfactory receptor neurons (not expressing ACE2) themselves or whether other cells such as supporting cells are damaged in the pathological process (70).

Furthermore, viruses can cause olfactory dysfunction by penetrating into the CNS and damaging brain regions that are components of the olfactory system, such as the olfactory bulb, the pirifom cortex, amygdala, the olfactory tubercle and more.

In a rodent study, Influenza A virus administered to mice intra-nasally was incorporated into the olfactory receptor neurons (ORNs) and traveled transneuronally (21, 71). As mentioned in the previous chapter, coronavirus was detected in the brain and the CSF (51–54). In particular, when the occurrence of anosmia does not temporarily concur with the symptoms of rhinitis or anosmia persists long after, it is to be concluded that neurological damage has occurred.

Current treatment options for PVOD imply the use of corticosteroids which are counter-indicated in patients presenting with anosmia, notably in the absence of known head trauma or allergic symptoms (72, 73). Another promising avenue of treatment for non-sino-nasal forms of olfactory dysfunction, such as PVOD, is olfactory training since repeated exposure to odors over a period of time seems to have a sensitizing effect and lower the threshold for tested odors (74). A multicenter double-blind study demonstrated that smell training in PVOD patients improved olfactory function significantly more than expected by spontaneous recovery (9, 73).

## Hyposmia in Allergic Rhinitis

Allergies often lead to allergic rhinitis, a type of inflammation in the nasal cavity, which occurs when the immune system overreacts to allergens in the air, symptoms of which include blocked airways and nasal discharge.

Inhalation of harmful environmental agents often damages the olfactory mucosa, triggering an immune response (75). Several animal models of human chronic rhinosinusitis show inflammatory responses in the nasal cavity, as well as general pathology of the olfactory epithelium that includes mast cell and eosinophilic infiltration, systemic inflammation and altered levels of inflammatory cytokines in the brain, concomitantly with the infiltration of macrophages and lymphocytes (76, 77). In a mouse model of irreversible AR-induced smell loss, CD45 staining showed an infiltration of leukocytes at the olfactory mucosa which negatively correlated with olfactory neuron number (78).

Allergic rhinitis (AR) is associated with a loss of smell, and 23–48% of AR patients present smell deficits (79, 80). One study showed that the longer allergic symptoms are experienced, the higher the probability for olfactory dysfunction (81). The presence of olfactory dysfunction seems to increase with the severity and duration of the disease. Data indicate that the frequency and severity of olfactory dysfunction increases in patients with persistent AR compared with patients with seasonal AR (82).

It was also determined that olfactory dysfunction was associated with nasal-sinus disease (measured by olfactory cleft visibility, and nasal airway obstruction) (83). Following corticoid treatment, rhinitis symptoms (e.g., mucosal thickening, polypoid changes) decreased significantly for all patients in the study. While olfactory function likewise improved significantly for 59% of the patients, the sense of smell in the remaining 41% did not change (84). Furthermore, patients with severe rhinitis are at increased risk to attract repeated respiratory tract infections, which then lead to damage to the olfactory epithelium and causes further olfactory dysfunction (85).

There is limited and contradictory evidence that antihistamines improve olfactory function in AR (86, 87). Slightly more encouraging data shows that the use of topical steroids is beneficial, especially in patients with seasonal AR and often as combined treatment of steroids and antihistamines [for a review see (88)]. In a mouse model of allergic rhinitis, it was shown that when olfaction is severely disturbed (mice take significantly longer to detect hidden food pellets than control mice), damage in the epithelia was found. Intranasal steroids were shown to revert the dysfunction and epithelial damage (89). Recent approaches in individualized immunotherapy to treat AR have also been effective in reducing the hyposmia symptoms (90–92).

Chronic rhinosinusitis with nasal polyps (CRSwNP) is an important clinical entity diagnosed by the presence of both subjective and objective evidence of chronic sinonasal inflammation. About 25% of patients with chronic rhinosinusitis develop CRSwNP (93) In the “SINUS” 24%2 study Dupilumab, a human monoclonal antibody that blocks a shared receptor component of interleukin 4 and 13 improved the sense of smell in patients with severe CRSwNP (94).

## Anosmia Induced by Drugs

A vast number of drugs from all pharmacological categories impair both taste and smell function and do so commonly. It is reported that 50% of the top 100 drugs in the United States have the potential to induce chemosensory complaints and side effects (95). Smell and taste disorders are also among the many side effects of chemo- and radiotherapy. Although direct radionecrosis of the salivary glands and the taste buds might explain the chemosensory problems after radiotherapy, the olfactory and gustatory complaints seen after chemotherapy remain unexplained. The patients reporting olfactory symptoms rarely complain about qualitative olfactory disorders such as parosmia or phantosmia.

Polypharmacy is a well-known prominent aspect of global medical practice and a growing concern in an aging society. Therefore, it is a growing concern that a significant number of chemosensory disturbances are a consequence of drug–drug interactions from polypharmacy rather than intake of a single drug (96). When two drugs are taken one drug can alter the bioavailability and/or pharmacological effects of a co-administered drug. In a study of elderly cardiovascular patients, those taking the greatest number of medications had the largest smell losses as well as the most complaints of altered taste.

Drugs that were found to interfere with the ability to smell or taste are for example certain antibiotics, anti-inflammatory drugs, CNS agents such as antidepressants or gastrointestinal drugs, as reviewed in (97).

Impairment may require discontinuation of drug administration. The inhibition of taste receptor is primarily due to drug induced inactivation of receptor function and impairment of receptor binding; Gs protein function; inositol trisphosphate function; as well as abnormal channel activity. Termination of drug therapy is commonly associated with termination of taste/smell dysfunction, but occasionally effects persist and require specific therapy to alleviate symptoms (98).

Furthermore is the chronic abuse of recreational drugs associated with hyposmia and defects in olfaction, especially with cocaine consumption. The mechanism of the toxicity of chronic cocaine abuse apparently involves impairment of calcium-mediated impulse transmission to the olfactory bulb from the chemosensory olfactory neurons. Anosmia induced by cocaine is thought to be mediated by actual infarction of the olfactory mucosa (99, 100).

## Anosmia in Aging and Age-Related Neurodegenerative Disease

Our sense of smell gets worse as we age. Ten percentage of people over 65 years have some form of olfactory dysfunction, ranging from mild loss to anosmia (101, 102), while in the age bracket of over 80 years, 62–80% of persons are affected (103). Men are stronger afflicted than women (104).

As we age, the pool of basal stem cells in the epithelium that is used to replenish dying olfactory neurons, is diminished, preventing a regeneration of such neurons and leading to a reduced thickness and function of the epithelium (9). In support, it has been shown that patients with congenic anosmia present an absent or atrophic olfactory epithelium in nasal mucosa (105). Immune cells and cytokines in the olfactory mucosa can play important roles in degeneration of olfactory neurons. It was suggested that an inflammatory process, comparable to those occurring after bulbectomy or after inhalation environmental toxicants, is in place, whereby lymphocytes, macrophages, and eosinophils release inflammatory mediators that up-regulate pro-apoptotic enzymes, exerting toxicity to olfactory neurons (75, 106).

Furthermore, with progressive age, central structures that are involved in olfactory perception, such as piriform cortex, amygdala, the entorhinal cortex and parts of the cerebellum are also less activated, as shown by fMRI (107). The size of the olfactory bulb decreases, reflecting a generalized atrophy caused by age (108).

Neurodegenerative diseases are an immense burden to our health systems and their prevalence is still projected to grow with an increasingly aging population worldwide. In this patient group, the prevalence of olfactory dysfunction is disproportionally higher than what would be predicted through the process of aging alone. Approximately 90% of patients with early-stage Parkinson's and 85% of patients with early-stage Alzheimer's disease experience olfactory dysfunction, as measured by psychophysical and electrophysiological tests (109, 110).

Interestingly, over the years, a correlation between olfactory dysfunction and neurodegenerative disorders has emerged (107, 111) that is particularly relevant for the two major diseases, Parkinson's and Alzheimer's disease. One striking phenomenon is that olfactory dysfunction precedes the onset of motor or cognitive symptoms in Parkinson's disease (PD) and Alzheimer's disease (AD) by several years. Thus, olfactory dysfunction has received attention as a potential early biomarker for Parkinson's (112), Alzheimer's disease (113) and Lewy body dementia (95, 114). It is obvious that earlier detection of PD/AD would enable the application of potential preventative disease-modifying treatment strategies.

## Parkinson's Disease

Hyposmia is common in idiopathic PD, i.e., the form of the disease of unknown etiology, that has not been linked to mutations in one of the Parkinson's disease genes, occurring generally 4–6 years before the onset of motor symptoms, and is being discussed as early biomarker, especially when combined with other early symptoms such as depression and or REM sleep behavior disorder. In contrast, complete anosmia occurs more rarely (115). As seen with other conditions, subjective assessment of olfactory dysfunction does not equal the measured outcomes since up to 72% of PD patients with olfactory dysfunction are unaware of it (116, 117). All qualities of olfactory function can be impaired by PD, from elevated threshold detection to reduced odor detection, discrimination and identification (109, 111, 118).

PD is characterized by a loss of midbrain dopaminergic (DA) neurons of the Substantia Nigra pars compacta (SNpc) and the appearance of α-synuclein inclusions, termed Lewy bodies. These midbrain DA neurons project to the caudate putamen and the cortex where they regulate, amongst others, movement and motor coordination. Interestingly, DA is also produced locally in the olfactory bulb by interneurons of the periglomerular layer (119, 120), while the olfactory bulb also receives DAergic input from the midbrain (121). The DA interneurons modulate olfactory abilities (122) and exhibit high plasticity in response to odor deprivation which leads to a reduction in cell number (119, 123, 124). It was found that the number of DAergic interneurons is unaltered in patients vs. a control group which excludes the hypothesis that hyposmia is mediated by an altered number of DA interneurons (125).

Studies assessing activity and volume of brain structures yielded differences in PD patients in comparison to the age-matched groups: In some but not all studies the volume of the olfactory bulb has been found reduced in PD (126, 127). Furthermore, MRI studies revealed pathological process in the nervous tissue of the olfactory tract of early PD patients (128). PD patients with anosmia further showed abnormal structural integrity in the central olfactory structures compared to PD patients without olfactory dysfunction or healthy controls (111, 129). FMRI studies show reduced neuronal activity of the amygdala and hippocampus and decreased functional connectivity in the primary olfactory cortices as well as the secondary olfactory structures compared with controls in PD patients during olfactory stimulation (130). EEG studies support the hypothesis that a decline of central brain networks is a causal factor for olfactory loss in PD (129) indicating that at onset of anosmia the pathology has already reached the CNS.

Another indicator for the involvement of the olfactory system in Parkinson's is the finding that α-synuclein aggregates were found across the central olfactory system, including the anterior olfactory nucleus, the cortical nucleus of the amygdala, the piriform cortex, the olfactory tubercle, the entorhinal cortex, and the orbitofrontal cortex prior to other regions, suggesting that the olfactory system might be particularly vulnerable, early in the disease (131, 132). In mice, the propagation in the brain of preformed fibrillar assemblies of recombinant α-synuclein was paralleled by a progressive reduction in olfactory function (133). Braak and colleagues reported that the expression of Lewy bodies begins in the olfactory bulb, the anterior olfactory nucleus and dorsal motor nucleus of the vagus nerve (Stage 1) and then advances to the raphe nuclei, traveling up the brain stem (pontine tegmentum) (stage 2) to reach the midbrain with SNpc DAergic neurons as well as the Nucleus of Meynert and cholinergic neurons in the basal forebrain (stage 3) (134).

These findings were developed into a hypothesis termed the vector hypothesis of PD in which an unknown pathogen, toxin or trigger may enter the central nervous system through the olfactory system (or via the stomach/vagus nerve or even via a succession of events starting in the olfactory bulb and reaching the enteric nervous system via the amygdala and stria terminalis) (21). Recent work in rodents where α-synuclein fibrils were micro-injected into the olfactory bulb, demonstrate that the pathology can spread to the substantia nigra and other regions involved in later stages of PD (132). The progressive development of α-synucleopathy was coupled to the emergence of specific olfactory deficits (133).

Importantly, direct connections are present between central olfactory structures and the substantia nigra. It was shown that intranasally administered influenza virus caused selective decreases of dopamine neurons in the substantia nigra of mice (75). Further, a reduced intrinsic integrity of the substantia nigra in patients with unexplained smell loss support the PD at-risk status of these patients (135). Another strengthening argument for the vector hypothesis stems from the finding that the cortical nucleus of the amygdala, which receives input from primary olfactory bulb projections, shows more α-synuclein pathology and neuronal loss than other nuclei of the amygdala (136). The loss of volume in the amygdala and the piriform cortex inversely correlates with olfactory deficits suggesting that cell loss in these regions could contribute to the functional deficits (132).

In addition to α-synuclein pathology, tau pathology has also been found in the anterior olfactory nucleus (AON) in PD (132, 137). Of the subcohort of patients with progressive supranuclear palsy and corticobasal degeneration, parkinsonian disorders that had an intact sense of smell, tau aggregates in the AON were also missing, pointing toward a possible correlation of tau pathology and olfactory impairment in PD (138).

In support of the reliability of anosmia as early biomarker in PD, it was shown that marked changes in olfactory threshold and odor discrimination correlated with a more rapid disease progression (139). A correlation of anosmia with reduced imaging of dopamine transporter (DAT), a biomarker that is located at the pre-synapse of SNpc DA neurons, was shown (112). In a prospective study, using olfactory testing in clinically unaffected first-degree relatives of PD patients showed that all of the hyposmic individuals with abnormal DAT imaging at baseline developed PD within 5 years (140). In longitudinal studies, the presence of severe olfactory dysfunction at diagnosis was able to predict cognitive decline, while normosmic patients with normal cognition remained cognitively stable for years (141, 142).

One study has also identified smell dysfunction in cases of corticobasal syndrome and frontotemporal dementia, urging caution for a differential diagnosis of parkinsonism based on hyposmia, however, the participant number in this study was low (143).

When it comes to genetically caused PD, the correlation with olfactory dysfunction appears complex: Patients with the A53T mutation of the α-synuclein gene exhibit hyposmia, but not patients with the α-synuclein E46K mutation (144, 145). Mutations in the Parkin gene are the most common cause for genetic PD, and yet in these patients no olfactory symptoms are present (146) while patients with mutations in *PINK1* present olfactory dysfunction (147). Finally, in patients with mutations in LRRK2, hyposmia is present but less frequent than in idiopathic PD. A meta-analysis determined that 51% of patients with the G2019S mutation patients exhibited significant olfactory deficits (148, 149). Therefore, other genetic or environmental factors, yet to be identified, must be involved for this phenotype to be penetrant.

Epidemiological studies have indicated an inverse association between smoking and PD (150, 151). PD risk is lowest among subjects with the longest duration of smoking, the greatest lifetime and/or daily dose of smoking, and, in past smokers, the fewest years since quitting (151). On the other hand, olfactory function was less attenuated in current cigarette smokers with PD than in non-smokers with PD (151, 152). Amongst PD patients, smokers scored significantly better in smell tests than non-smokers, while in the healthy control group no difference between smokers and non-smokers was observed in (152). This is in contrast to findings in the overall population where smoking was associated with a reduced sense of smell, and heavy smoking (> 20 cigarettes per day) also negatively affected the sense of taste (153). A meta-analysis of the effect of smoking on olfactory function, yielded a more differentiated outcome, by which former smokers performed similarly to non-smokers while active had a reduced smell scores, indicating that the deleterious effect of smoking is reversible (154). It has not been studied if the above-mentioned functional interaction between nicotinic receptors and ACE2 is relevant for this effect, in healthy control or PD patients.

In the olfactory bulb, dopamine receptors of the D1 and D2 type are present. In rats, local or central D2 agonism reduces olfactory function, while D1 agonism enhances it (122), suggesting an inhibitory and stimulatory function for D2 and D1 receptors, respectively. L-DOPA treatment, the gold standard treatment that almost all PD patients receive, has been shown to not affect olfactory performance (115). In contrast, deep brain stimulation in the subthalamic nucleus resulted in a partial rescue olfactory processing (155).

## Alzheimer's Disease

Olfactory dysfunction is an early symptom of AD and approximately 85% of patients with early-stage AD experience olfactory dysfunction (110). Olfactory deficits occur on all levels: odorant detection and detection threshold, identification, recognition and discrimination, as well as odor memory are affected in AD, with odor identification being particularly reduced (156). In cognitively normal older individuals, worse odor identification has been associated with increased cortical amyloid, and with neurofibrillary pathology in the entorhinal cortex and hippocampus (113).

In humans and in mouse models, amyloid and tau deposits have been found throughout the olfactory pathways, including temporal piriform cortex at earlier stages of the disease before other regions such as the entorhinal cortex or CA1 region of the hippocampus, both olfactory projection areas, are affected (157–159). Functional MRI studies have shown reduced blood oxygenation in the olfactory cortex of early-stage AD patients (160).

In the olfactory bulb, the major pathophysiological hallmarks are abnormally phosphorylated tau and neurofibrillary tangles, however amyloid plaques have also been found (157). Mouse models of Alzheimer s disease also exhibit an olfactory loss phenotype (161, 162), and in mouse models Notch and Reelin pathways have been implicated (163, 164). Due to the high variance of Aβ, a clear correlation between Aβ and anosmia has not been established and contradictory results have been published (161, 165, 166), in contrast, neurofibrillary tangles in the olfactory bulb, the entorhinal cortex and the CA1 region are correlates with olfactory dysfunction (167, 168).

The loss of the cholinergic system, in particular the nucleus of Meynert, plays an important role in AD and cholinesterase inhibitors are recommended for us in people with mild to moderate AD (169) to delay the loss of brain function. Interestingly, in a small, non-blinded study, it was shown that use of a cholinesterase inhibitor, donepezil, correlated with an improvement in olfactory function of AD patients (170). This data should be easily validated in large retrospective studies, given that cholinesterase inhibitors are widely used.

The “vector hypothesis of PD” described above has also been applied to the pathogenesis of AD but is more ambiguous since it is less clear whether AD pathology first appears within the peripheral olfactory system or in central (olfaction-related) brain regions (21). According to Braak, neurofibrillary tangles occur initially in the transentorhinal region. Plaques and tangles in the olfactory bulb and tract were detected at a lower density than in the amygdala and hippocampus (171).

For both PD and AD, the vector hypothesis has been questioned since it cannot explain the existence of genetic and familial forms of AD and PD and the lack of smell dysfunction in some 10% of idiopathic patients. However, the vector hypothesis does not preclude other pathological mechanisms from occurring that are possibly driven by unknown mutations, epigenetic mechanisms, or other determinants.

## Other Age-Related Neurodegenerative Disorders

### Huntington Disease

Huntington disease is associated with moderate hyposmia. Family members with a 50% risk of disease show no olfactory abnormalities, so it can be assumed that the olfactory changes begin at the same time as the motor and cognitive symptoms (172). In contrast, odor discrimination impairments in asymptomatic carriers of the Huntington disease gene were described (173).

### Amyotrophic Lateral Sclerosis (ALS)

Hyposmia has been described as one of the non-motor neuron symptoms in ALS, the most common age-related motor neuron disorder (174). ALS patients scored significantly lower on the University of Pennsylvania Smell Identification Test (UPSIT) (175), with experimental setups using the “Sniffin' Sticks” (176, 177), and with the odor stick identification test for Japanese (OSIT-J) (178). One of these studies suggested that mild impairment of olfaction preferably occurs in ALS patients with impaired respiratory function (177). In another study, hyposmia accumulated in ALS subjects which also suffered from cognitive or behavioral dysfunction (176), and in the third study no specific ALS subset could be identified (175). As the number of ALS patients analyzed was low in these studies, a systematic analysis of a larger cohort of ALS patients will be needed for validating whether hyposmia is a prognostic marker for a specific subset of ALS. This could be helpful for a more adequate treatment of affected people.

In motor neurons, ubiquitylated protein inclusions composed of the RNA-binding protein TDP-43 are pathological hallmarks of ALS. Of note, TDP-43-positive inclusion could also be observed in hippocampus, in the primary olfactory center, and, to a lower extent, in the olfactory bulb (178, 179). Thus, it is tempting to speculate that impairment of the olfactory knowledge in the brain is the reason for odor loss in ALS, rather than defective olfactory perception. Table 1 characterizes the characteristics of smell and taste pathologies including treatment options and Table 2 presents an overview of the known and presumed mechanisms underlying the the pathology of smell loss.

**Table 1:** Major smell and taste-altering pathologies and characteristics.

**Characteristics**	**Viral-induced**		**Allergies**	**Drug-induced**	**Neurdegenerative diseases**				
	**Covid-19**	**Infliuenza and other viruses**	**Allergic rhinitis**	**Various**	**Normal ageing**	**Parkinson's disease**	**Alzheimer's disease**	**Huntington**	**ALS**
Loss of olfaction	Yes	Yes	Yes	Yes	Yes	Yes	Yes	Moderate	Yes
Loss of taste	Yes	Yes	Yes	Yes	Yes, but mainly caused by reduced smell	Yes	Yes, but less prominent	Not described, but dry mouth affects appetite	Yes
Onset	Rapid and early	Rapid	Slow		Slow	Early symptom	Early symptom	At time of disease onset	Shortly after disease onset
Regression	Rapid in most cases	Slow (over years) but up to 66% reocever	Frequent but slow and can re-occur	Usually occurs after termination of drug	No	No	No	No	No
Occurrence	Up to 70%	Less than 1%	Up to 60%		10% over age of 65 years	Up to 90%	Up to 85%	No	Rarely
Gender and age factors	In younger, less clinically afflicted patients	45–65 years, women more affected	30–60 years		Age, males more affected	Smokers scored significantly better in smell tests	No age or gender differences found	Not known	Age positvely corelates with loss of function
Treatment	Cortico-steroids not recommended, under investigation	Olfactory training, cortico-steroids	Cortico-steroids, anti-histamines, immuno-therapy	Termination of drugs	None	Frequent exposure training, deep brain stimulatiom, D1 agonism	Frequent exposure training,cholin-esterase inhibitor	None	None

*Sources: mainly based on Doty et al. (21), Huettenbrink et al. (9), Marin et al. (111), Hummel et al. (74), Watts et al. (88), Henkin et al. (98)*.

**Table 2:** Summarizing known and presumed mechanisms underlying the pathology of smell loss in the various human conditions and disorders.

**Type of dysosmia**	**Underlying mechanism**	**References**
Covid-19	under investigation presumed neurological damage due to peripheral central neuroinvasion of virus	
Influenza	Dysosmia during acute infection: olfactory loss due to swelling of nasal mucosa airway obstruction post-viral stage dysosmia: presumably neurological damage is at play since viruses were detected in CSF brain olfacory receptor neurons.	(43–46, 59)
Allergic rhinitis	Nasal airway obstruction caused by inflammation since corticoid treatment reduces the smell defect accumulation of CD45+ leukocytes during inflammatory process	(69, 75)
Drug-induced	Mechanism can be many fold depends on specific drug. Certain drugs were shown to affect signaling capacity of olfacory receptor neurons.	(89–91)
Ageing	central peripheral mechanisms: atrophy of olfactory epithelium reduced activity of central olfaction-related brain structures	(96, 98)
Parkinson's disease	Neurological mechanism: a-synuclein pathology was found in the central olfactory system (anterior olfactory nucleus cortical nucleus of the amygdala piriform cortex olfactory tubercle the entorhinal cortex others). However causality still needs to be established. Further a decline of central brain networks may cause olfactory loss in PD.	(120, 122, 123)
Alzheimer's disease	Neurological mechanism: Amyloid tau deposits were detected in olfactory pathways (temporal piriform cortex entorhinal cortex CA1). However causality still needs to be established.	(147–149, 157, 158)
Huntington's disease	Unknown	
ALS	Unknown	

### Current Treatment Options for Anosmia and Hyposmia

Long lasting or even the permanent loss of olfactory function markedly reduces the quality of life. Therefore, several therapeutic attempts have been applied to accelerate the recovery or to increase the ability to smell, including pharmacological intervention, surgical treatment, and olfactory training.

Corticosteroid have been used as a pharmacological approach to treat patients suffering from olfactory dysfunction upon upper respiratory infections, chronic rhinosinusitis and other reasons (180). Although these approaches frequently improve olfaction, the effects often disappear after ceasing treatment. Inhibiting phosphodiesterase activity using the non-specific phosphodiesterase inhibitor theophylline, prolongs the intracellular signaling cascade augmenting odor perception. Thus, olfactory sensitivity is increased applying this pharmacological approach (181). However, in most cases lasting clinical efficacy can also not be reached with this option.

Surgical treatment as treatment option for smell loss has been extensively studied for chronic rhinosinusitis. However, it remains difficult to predict the improvement of olfactory dysfunction upon surgery (180).

Notably, the capacity of olfactory receptor neurons to regenerate can be modulated by the exposure to certain odors for a couple of weeks. This olfactory training appears to work in patients with olfactory dysfunction due to multiple etiologies, including infections, trauma, Parkinson's disease and unknown reasons (idiopathic anosmia) (182). Especially people affected by postviral olfactory dysfunctions benefit from olfactory training (183). Therefore, this approach might be suitable for people suffering from permanent total or partial loss of olfactory function due to Covid-19. The molecular and cellular mechanisms behind the beneficial effects of olfactory training are poorly understood, but neuroplasticity could play a crucial role here (184).

## Conclusions

Olfactory dysfunction can be caused by a multitude of agents and in the process of various pathologies. Of note, hyposmia and anosmia are reliable early symptoms in different pathological situations, ranging from viral infections, including SARS-CoV2, to common neurodegenerative disorders, including Alzheimer's and Parkinson's diseases. In addition to its valuable function as a potential diagnostic biomarker in these diseases, the olfactory system could also be the entry point for viruses and toxins to the brain. This could lead to infections and harmful neuroinflammatory reactions in the central nervous system. Clearly, viruses were shown to be transported along synaptic connections from the peripheral olfactory epithelium, into the CNS, where they first target regions that are part of the olfactory system (olfactory bulb, amygdala and others) to subsequently reach other structures that will trigger the development of disease-specific symptoms, such as motor and cognitive symptoms, or epilepsies. It is of utmost importance to determine, on a cellular and molecular level, the mechanisms underlying the olfactory dysfunction and the potential detrimental propagation along the olfactory pathway. This will enable us to develop novel tools to interfere with disease progression.

## Author Contributions

This manuscript was written through contributions from all authors. HR wrote and conceptualized paper. RB, DL, WK, CK, and AH contributed to manuscript writing and to the preparation of figures. All authors have given approval to the final version of the manuscript.

## Conflict of Interest

The authors declare that the research was conducted in the absence of any commercial or financial relationships that could be construed as a potential conflict of interest.

## References

[1] Deems DA Doty RL Settle RG Moore-Gillon V Shaman P Mester AF. Smell and taste disorders, a study of 750 patients from the university of pennsylvania smell and taste center. Arch Otolaryngol Head Neck Surg. (1991) 117:519–28. 10.1001/archotol.1991.01870170065015 2021470

[2] Mirza RS Green WW Connor S Weeks AC Wood CM Pyle GG. Do you smell what I smell? Olfactory impairment in wild yellow perch from metal-contaminated waters. Ecotoxicol Environ Saf. (2009) 72:677–83. 10.1016/j.ecoenv.2008.10.001 19108892

[3] Bramerson A Nyman J Nordin S Bende M. Olfactory loss after head and neck cancer radiation therapy. Rhinology. (2013) 51:206–9. 10.4193/Rhin12.120 23943726

[4] Mirza N Machtay M Devine PA Troxel A Abboud SK Doty RL. Gustatory impairment in patients undergoing head and neck irradiation. Laryngoscope. (2008) 118:24–31. 10.1097/MLG.0b013e318155a276 17975512

[5] Fischer DJ Epstein JB. Management of patients who have undergone head and neck cancer therapy. Dent Clin North Am. (2008) 52:39–60. 10.1016/j.cden.2007.09.004 18154864

[6] Briner HR Simmen D Jones N. Impaired sense of smell in patients with nasal surgery. Clin Otolaryngol Allied Sci. (2003) 28:417–9. 10.1046/j.1365-2273.2003.00735.x 12969343

[7] Bromley SM. Smell and taste disorders: a primary care approach. Am Fam Physician. (2000) 61:427–36. 10670508

[8] Bartoshuk LM Beauchamp GK Chemical senses. Annu Rev Psychol. (1994) 45:419–49. 10.1146/annurev.ps.45.020194.002223 8135507

[9] Huttenbrink KB Hummel T Berg D Gasser T Hahner A. Olfactory dysfunction: common in later life and early warning of neurodegenerative disease. Dtsch Arztebl Int. (2013) 110:1–7. 10.3238/arztebl.2013.0001 23450985 PMC3561743

[10] Boesveldt S Postma EM Boak D Welge-Luessen A Schopf V Mainland JD. Anosmia-a clinical review. Chem Senses. (2017) 42:513–23. 10.1093/chemse/bjx025 28531300 PMC5863566

[11] Kollndorfer K Reichert JL Bruckler B Hinterleitner V Schopf V. Self-esteem as an important factor in quality of life and depressive symptoms in anosmia: a pilot study. Clin Otolaryngol. (2017) 42:1229–34. 10.1111/coa.12855 28236363 PMC5724690

[12] Croy I Nordin S Hummel T. Olfactory disorders and quality of life–an updated review. Chem Senses. (2014) 39:185–94. 10.1093/chemse/bjt072 24429163

[13] Buck LB. Olfactory receptors and odor coding in mammals. Nutr Rev. (2004) 62(11 Pt 2):S184–8. 10.1301/nr.2004.nov.S184-S188 15630933

[14] Brennan PA. 50 years of decoding olfaction. Brain Neurosci Adv. (2018) 2:2398212818817496. 10.1177/2398212818817496 32166167 PMC7058195

[15] Olender T Nativ N Lancet D. HORDE: comprehensive resource for olfactory receptor genomics. Methods Mol Biol. (2013) 1003:23–8. 10.1007/978-1-62703-377-0_2 23585031

[16] Kleene SJ Gesteland RC. Dissociation of frog olfactory epithelium with N-ethylmaleimide. Brain Res. (1981) 229:536–40. 10.1016/0006-8993(81)91018-0 6975646

[17] Bushdid C Magnasco MO Vosshall LB Keller A. Humans can discriminate more than 1 trillion olfactory stimuli. Science. (2014) 343:1370–2. 10.1126/science.1249168 24653035 PMC4483192

[18] Nef P. How we smell: the molecular and cellular bases of olfaction. News Physiol Sci. (1998) 13:1–5. 10.1152/physiologyonline.1998.13.1.1 11390750

[19] Nagayama S Homma R Imamura F. Neuronal organization of olfactory bulb circuits. Front Neural Circuits. (2014) 8:98. 10.3389/fncir.2014.00098 25232305 PMC4153298

[20] Djupesland PG Messina JC Mahmoud RA. The nasal approach to delivering treatment for brain diseases: an anatomic, physiologic, and delivery technology overview. Ther Deliv. (2014) 5:709–33. 10.4155/tde.14.41 25090283

[21] Doty RL. The olfactory vector hypothesis of neurodegenerative disease: is it viable? Ann Neurol. (2008) 63:7–15. 10.1002/ana.21327 18232016

[22] Zanusso G Ferrari S Cardone F Zampieri P Gelati M Fiorini M. Detection of pathologic prion protein in the olfactory epithelium in sporadic creutzfeldt-jakob disease. N Engl J Med. (2003) 348:711–9. 10.1056/NEJMoa022043 12594315

[23] Cowart BJ. Taste dysfunction: a practical guide for oral medicine. Oral Dis. (2011) 17:2–6. 10.1111/j.1601-0825.2010.01719.x 20796233

[24] Berridge KC Fentress JC. Trigeminal-taste interaction in palatability processing. Science. (1985) 228:747–50. 10.1126/science.3992242 3992242

[25] Suzuki M Saito K Min WP Vladau C Toida K Itoh H. Identification of viruses in patients with postviral olfactory dysfunction. Laryngoscope. (2007) 117:272–7. 10.1097/01.mlg.0000249922.37381.1e 17277621 PMC7165544

[26] Lechien JR Cabaraux P Chiesa-Estomba CM Khalife M Hans S Calvo-Henriquez C. Objective olfactory evaluation of self-reported loss of smell in a case series of 86 COVID-19 patients. Head Neck. (2020) 42. 10.1101/2020.05.03.20088526 32437033 PMC7280665

[27] Hopkins C Surda P Whitehead E Kumar BN Early recovery following new onset anosmia during the COVID-19 pandemic - an observational cohort study. J Otolaryngol Head Neck Surg. (2020) 49:26 10.1186/s40463-020-00423-8 32366299 PMC7196882

[28] Melley LE Bress E Polan E. Hypogeusia as the initial presenting symptom of COVID-19. BMJ Case Rep. (2020) 13:e236080. 10.1136/bcr-2020-236080 32404376 PMC7228456

[29] Lechien JR Chiesa-Estomba CM Hans S Barillari MR Jouffe L Saussez S. Loss of smell and taste in 2013 European patients with mild to moderate COVID-19. Ann Intern Med. (2020). 10.7326/M20-2428. [Epub ahead of print]. 32449883 PMC7505100

[30] Mercante G Ferreli F De Virgilio A. Prevalence of taste and smell dysfunction in coronavirus disease 2019. JAMA Otolaryngol Head Neck Surg. (2020) 146:723–8. 10.1001/jamaoto.2020.1155 32556070 PMC7303892

[31] Yan CH Faraji F Prajapati DP Boone CE DeConde AS. Association of chemosensory dysfunction and Covid-19 in patients presenting with influenza-like symptoms. Int Forum Allergy Rhinol. (2020) 10:806–13. 10.1002/alr.22579 32279441 PMC7262089

[32] Luers JC Klussmann JP Guntinas-Lichius O. (The Covid-19 pandemic and otolaryngology: what it comes down to?). Laryngorhinootologie. (2020) 99:287–91. 10.1055/a-1095-2344 32215896

[33] Lechien JR Place S Chiesa-Estomba CM Khalife M De Riu G Vaira LA. The prevalence of SLS in severe COVID-19 patients appears to be lower than previously estimated in mild-to-moderate COVID-19 forms. Pathogens. (2020) 9:627. 10.3390/pathogens9080627 32752123 PMC7460289

[34] Beltran-Corbellini A Chico-Garcia JL Martinez-Poles J Rodriguez-Jorge F Natera-Villalba E Gomez-Corral J Acute-onset smell and taste disorders in the context of COVID-19: a pilot multicentre polymerase chain reaction based case-control study. Eur J Neurol. (2020) 10.1111/ene.14359. [Epub ahead of print]. PMC7264557 32320508

[35] Klopfenstein T Kadiane-Oussou NJ Toko L Royer PY Lepiller Q Gendrin V. Features of anosmia in COVID-19. Med Mal Infect. (2020) 50:436–9. 10.1016/j.medmal.2020.04.006 32305563 PMC7162775

[36] Vabret N Britton GJ Gruber C Hegde S Kim J Kuksin M. Immunology of COVID-19: current state of the science. Immunity. (2020) 52:910–41. 10.1016/j.immuni.2020.05.002 32505227 PMC7200337

[37] Soter A Kim J Jackman A Tourbier I Kaul A Doty RL. Accuracy of self-report in detecting taste dysfunction. Laryngoscope. (2008) 118:611–7. 10.1097/MLG.0b013e318161e53a 18182967

[38] Adams DR Wroblewski KE Kern DW Kozloski MJ Dale W McClintock MK. Factors associated with inaccurate self-reporting of olfactory dysfunction in older US adults. Chem Senses. (2017) 42:223–31. 10.1093/chemse/bjw108 28007787 PMC6074942

[39] Shang J Ye G Shi K Wan Y Luo C Aihara H. Structural basis of receptor recognition by SARS-CoV-2. Nature. (2020) 581:221–4. 10.1038/s41586-020-2179-y 32225175 PMC7328981

[40] Li YC Bai WZ Hashikawa T. The neuroinvasive potential of SARS-CoV2 may play a role in the respiratory failure of COVID-19 patients. J Med Virol. (2020) 92:552–55. 10.1002/jmv.25728 32104915 PMC7228394

[41] Lechien JR Radulesco T Calvo-Henriquez CC Chiesa-Estomba CM Hans S Barilari MR ACE2 & TMPRSS2 expressions in head & neck tissues: a systematic review. Head Neck Pathol. (2020) 2020:1–11. 10.1007/s12105-020-01212-5 PMC7439628 32816230

[42] Moein ST Hashemian SMR Mansourafshar B Khorram-Tousi A Tabarsi P Doty RL. Smell dysfunction: a biomarker for COVID-19. Int Forum Allergy Rhinol. (2020) 10:944–50. 10.1002/alr.22587 32301284 PMC7262123

[43] Bilinska K Jakubowska P Von Bartheld CS Butowt R. Expression of the SARS-CoV-2 entry proteins, ACE2 and TMPRSS2, in cells of the olfactory epithelium: identification of cell types and trends with age. ACS Chem Neurosci. (2020) 11:1555–62. 10.1021/acschemneuro.0c00210 32379417 PMC7241737

[44] Brann DH Tsukahara T Weinreb C Lipovsek M Van den Berge K Gong B. Non-neuronal expression of SARS-CoV-2 entry genes in the olfactory system suggests mechanisms underlying COVID-19-associated anosmia. bioRxiv. (2020). 10.1101/2020.03.25.009084 32937591 PMC10715684

[45] Bohmwald K Galvez NMS Rios M Kalergis AM. Neurologic alterations due to respiratory virus infections. Front Cell Neurosci. (2018) 12:386. 10.3389/fncel.2018.00386 30416428 PMC6212673

[46] Baig AM Khaleeq A Ali U Syeda H. Evidence of the COVID-19 virus targeting the CNS: tissue distribution, host-virus interaction, and proposed neurotropic mechanisms. ACS Chem Neurosci. (2020) 11:995–8. 10.1021/acschemneuro.0c00122 32167747

[47] McGavern DB Kang SS. Illuminating viral infections in the nervous system. Nat Rev Immunol. (2011) 11:318–29. 10.1038/nri2971 21508982 PMC5001841

[48] Lochhead JJ Thorne RG. Intranasal delivery of biologics to the central nervous system. Adv Drug Deliv Rev. (2012) 64:614–28. 10.1016/j.addr.2011.11.002 22119441

[49] Dahm T Rudolph H Schwerk C Schroten H Tenenbaum T. Neuroinvasion and inflammation in viral central nervous system infections. Mediators Inflamm. (2016) 2016:8562805. 10.1155/2016/8562805 27313404 PMC4897715

[50] Chapman CD Frey WH 2nd Craft S Danielyan L Hallschmid M Schioth HB. Intranasal treatment of central nervous system dysfunction in humans. Pharm Res. (2013) 30:2475–84. 10.1007/s11095-012-0915-1 23135822 PMC3761088

[51] Arbour N Day R Newcombe J Talbot PJ. Neuroinvasion by human respiratory coronaviruses. J Virol. (2000) 74:8913–21. 10.1128/JVI.74.19.8913-8921.2000 10982334 PMC102086

[52] Hung EC Chim SS Chan PK Tong YK Ng EK Chiu RW. Detection of SARS coronavirus RNA in the cerebrospinal fluid of a patient with severe acute respiratory syndrome. Clin Chem. (2003) 49:2108–9. 10.1373/clinchem.2003.025437 14633896 PMC7108123

[53] Lau KK Yu WC Chu CM Lau ST Sheng B Yuen KY. Possible central nervous system infection by SARS coronavirus. Emerg Infect Dis. (2004) 10:342–4. 10.3201/eid1002.030638 15030709 PMC3322928

[54] Yeh EA Collins A Cohen ME Duffner PK Faden H. Detection of coronavirus in the central nervous system of a child with acute disseminated encephalomyelitis. Pediatrics. (2004) 113(1 Pt 1):e73–6. 10.1542/peds.113.1.e73 14702500

[55] Gandhi S Srivastava AK Ray U Tripathi PP. Is the collapse of the respiratory center in the brain responsible for respiratory breakdown in COVID-19 patients? ACS Chem Neurosci. (2020) 11:1379–81. 10.1021/acschemneuro.0c00217 32348111

[56] Desforges M Miletti TC Gagnon M Talbot PJ. Activation of human monocytes after infection by human coronavirus 229E. Virus Res. (2007) 130:228–40. 10.1016/j.virusres.2007.06.016 17669539 PMC7114174

[57] Stegelmeier AA van Vloten JP Mould RC Klafuric EM Minott JA Wootton SK. Myeloid cells during viral infections and inflammation. Viruses. (2019) 11:168. 10.3390/v11020168 30791481 PMC6410039

[58] Kabbani N Olds JL. Does COVID19 infect the brain? If so, smokers might be at a higher risk. Mol Pharmacol. (2020) 97:351–3. 10.1124/molpharm.120.000014 32238438 PMC7237865

[59] Sharer JD Leon-Sarmiento FE Morley JF Weintraub D Doty RL. Olfactory dysfunction in parkinson's disease: positive effect of cigarette smoking. Mov Disord. (2015) 30:859–62. 10.1002/mds.26126 25545729 PMC4439272

[60] RECOVERY Collaborative Group Horby P Lim WS Emberson JR Mafham M Bell JL Linsell L. Dexamethasone in hospitalized patients with Covid-19 - preliminary report. N Engl J Med. (2020). 10.1056/NEJMoa2021436. [Epub ahead of print]. 32678530 PMC7383595

[61] Veronese N Demurtas J Yang L Tonelli R Barbagallo M Lopalco P. Use of corticosteroids in coronavirus disease 2019 pneumonia: a systematic review of the literature. Front Med. (2020) 7:170. 10.3389/fmed.2020.00170 32391369 PMC7193030

[62] Potter MR Chen JH Lobban NS Doty RL. Olfactory dysfunction from acute upper respiratory infections: relationship to season of onset. Int Forum Allergy Rhinol. (2020) 10:706–12. 10.1002/alr.22551 32282136 PMC7262030

[63] Schwartz JS Tajudeen BA Kennedy DW. Diseases of the nasal cavity. Handb Clin Neurol. (2019) 164:285–302. 10.1016/B978-0-444-63855-7.00018-6 31604553 PMC7151940

[64] Blaas D. Viral entry pathways: the example of common cold viruses. Wien Med Wochenschr. (2016) 166:211–26. 10.1007/s10354-016-0461-2 27174165 PMC4871925

[65] Sugiura M Aiba T Mori J Nakai Y. An epidemiological study of postviral olfactory disorder. Acta Otolaryngol Suppl. (1998) 538:191–6. 10.1080/00016489850182918 9879419

[66] Sullender WM. Respiratory syncytial virus genetic and antigenic diversity. Clin Microbiol Rev. (2000) 13:1–15. 10.1128/CMR.13.1.1 10627488 PMC88930

[67] Lemaire D Barbosa T Rihet P. Coping with genetic diversity: the contribution of pathogen and human genomics to modern vaccinology. Braz J Med Biol Res. (2012) 45:376–85. 10.1590/S0100-879X2011007500142 22030866 PMC3854287

[68] Flanagan CE Wise SK DelGaudio JM Patel ZM. Association of decreased rate of influenza vaccination with increased subjective olfactory dysfunction. JAMA Otolaryngol Head Neck Surg. (2015) 141:225–8. 10.1001/jamaoto.2014.3399 25590362

[69] Akerlund A Bende M Murphy C. Olfactory threshold and nasal mucosal changes in experimentally induced common cold. Acta Otolaryngol. (1995) 115:88–92. 10.3109/00016489509133353 7762392

[70] Welge-Lussen A Wolfensberger M. Olfactory disorders following upper respiratory tract infections. Adv Otorhinolaryngol. (2006) 63:125–32. 10.1159/000093758 16733337

[71] Aronsson F Robertson B Ljunggren HG Kristensson K. Invasion and persistence of the neuroadapted influenza virus A/WSN/33 in the mouse olfactory system. Viral Immunol. (2003) 16:415–23. 10.1089/088282403322396208 14583155

[72] Doty RL Treatments for smell and taste disorders: a critical review. Handb Clin Neurol. (2019) 164:455–79. 10.1016/B978-0-444-63855-7.00025-3 31604562

[73] Miwa T Ikeda K Ishibashi T Kobayashi M Kondo K Matsuwaki Y. Clinical practice guidelines for the management of olfactory dysfunction - secondary publication. Auris Nasus Larynx. (2019) 46:653–62. 10.1016/j.anl.2019.04.002 31076272

[74] Hummel T Rissom K Reden J Hahner A Weidenbecher M Huttenbrink KB. Effects of olfactory training in patients with olfactory loss. Laryngoscope. (2009) 119:496–9. 10.1002/lary.20101 19235739

[75] Imamura F Hasegawa-Ishii S. Environmental toxicants-induced immune responses in the olfactory mucosa. Front Immunol. (2016) 7:475. 10.3389/fimmu.2016.00475 27867383 PMC5095454

[76] Hasegawa-Ishii S Inaba M Umegaki H Unno K Wakabayashi K Shimada A. Endotoxemia-induced cytokine-mediated responses of hippocampal astrocytes transmitted by cells of the brain-immune interface. Sci Rep. (2016) 6:25457. 10.1038/srep25457 27149601 PMC4857737

[77] Baraniuk JN. Pathogenesis of allergic rhinitis. J Allergy Clin Immunol. (1997) 99:S763–72. 10.1016/S0091-6749(97)70125-8 9042069

[78] Liang C Yang Z Zou Q Zhou M Liu H Fan J. Construction of an irreversible allergic rhinitis-induced olfactory loss mouse model. Biochem Biophys Res Commun. (2019) 513:635–41. 10.1016/j.bbrc.2019.03.110 30981508

[79] Meltzer EO Jalowayski AA Orgel HA Harris AG. Subjective and objective assessments in patients with seasonal allergic rhinitis: effects of therapy with mometasone furoate nasal spray. J Allergy Clin Immunol. (1998) 102:39–49. 10.1016/S0091-6749(98)70053-3 9679846

[80] Cowart BJ Flynn-Rodden K McGeady SJ Lowry LD. Hyposmia in allergic rhinitis. J Allergy Clin Immunol. (1993) 91:747–51. 10.1016/0091-6749(93)90194-K 8454797

[81] Apter AJ Gent JF Frank ME. Fluctuating olfactory sensitivity and distorted odor perception in allergic rhinitis. Arch Otolaryngol Head Neck Surg. (1999) 125:1005–10. 10.1001/archotol.125.9.1005 10488987

[82] Rydzewski B Pruszewicz A Sulkowski WJ. Assessment of smell and taste in patients with allergic rhinitis. Acta Otolaryngol. (2000) 120:323–6. 10.1080/000164800750001189 11603799

[83] Soler ZM Hyer JM Karnezis TT Schlosser RJ. The olfactory cleft endoscopy scale correlates with olfactory metrics in patients with chronic rhinosinusitis. Int Forum Allergy Rhinol. (2016) 6:293–8. 10.1002/alr.21655 26718315 PMC4783185

[84] Mott AE Cain WS Lafreniere D Leonard G Gent JF Frank ME. Topical corticosteroid treatment of anosmia associated with nasal and sinus disease. Arch Otolaryngol Head Neck Surg. (1997) 123:367–72. 10.1001/archotol.1997.01900040009001 9109781

[85] Litvack JR Fong K Mace J James KE Smith TL. Predictors of olfactory dysfunction in patients with chronic rhinosinusitis. Laryngoscope. (2008) 118:2225–30. 10.1097/MLG.0b013e318184e216 19029858 PMC2652888

[86] Stuck BA Hummel T. Olfaction in allergic rhinitis: a systematic review. J Allergy Clin Immunol. (2015) 136:1460–70. 10.1016/j.jaci.2015.08.003 26409662

[87] Kalpaklioglu AF Kavut AB. Comparison of azelastine versus triamcinolone nasal spray in allergic and nonallergic rhinitis. Am J Rhinol Allergy. (2010) 24:29–33. 10.2500/ajra.2010.24.3423 20109317

[88] Watts AM Cripps AW West NP Cox AJ. Modulation of allergic inflammation in the nasal mucosa of allergic rhinitis sufferers with topical pharmaceutical agents. Front Pharmacol. (2019) 10:294. 10.3389/fphar.2019.00294 31001114 PMC6455085

[89] Jung AY Kim YH. Reversal of olfactory disturbance in allergic rhinitis related to OMP suppression by intranasal budesonide treatment. Allergy Asthma Immunol Res. (2020) 12:110–24. 10.4168/aair.2020.12.1.110 31743968 PMC6875474

[90] Tansuker D Coskun BU Ucal YO Sozen E Erdurak C Sakalli E. Effects of systemic immunotherapy on olfactory function in allergic rhinitis patients. J Craniofac Surg. (2014) 25:e339–43. 10.1097/SCS.0000000000000599 25006938

[91] Katotomichelakis M Riga M Tripsianis G Balatsouras D Kourousis C Danielides G. Predictors of quality of life improvement in allergic rhinitis patients after sublingual immunotherapy. Ann Otol Rhinol Laryngol. (2015) 124:430–6. 10.1177/0003489414565001 25539660

[92] Mun SJ Shin JM Han DH Kim JW Kim DY Lee CH. Efficacy and safety of a once-daily sublingual immunotherapy without escalation regimen in house dust mite-induced allergic rhinitis. Int Forum Allergy Rhinol. (2013) 3:177–83. 10.1002/alr.21098 23044892

[93] Stevens WW Schleimer RP Kern RC Chronic rhinosinusitis with nasal polyps. J Allergy Clin Immunol Pract. (2016) 4:565–72. 10.1016/j.jaip.2016.04.012 27393770 PMC4939220

[94] Bachert C Han JK Desrosiers M Hellings PW Amin N Lee SE. Efficacy and safety of dupilumab in patients with severe chronic rhinosinusitis with nasal polyps (LIBERTY NP SINUS-24 and LIBERTY NP SINUS-52): results from two multicentre, randomised, double-blind, placebo-controlled, parallel-group phase 3 trials. Lancet. (2019) 394:1638–50. 10.1016/S0140-6736(19)31881-1 31543428

[95] Schiffman SS Clark CM Warwick ZS. Gustatory and olfactory dysfunction in dementia: not specific to Alzheimer's disease. Neurobiol Aging. (1990) 11:597–600. 10.1016/0197-4580(90)90023-S 2280803

[96] Imoscopi A Inelmen EM Sergi G Miotto F Manzato E. Taste loss in the elderly: epidemiology, causes and consequences. Aging Clin Exp Res. (2012) 24:570–9. 10.3275/8520 22828477

[97] Schiffman SS. Influence of medications on taste and smell. World J Otorhinolaryngol Head Neck Surg. (2018) 4:84–91. 10.1016/j.wjorl.2018.02.005 30035266 PMC6051304

[98] Henkin RI. Drug-induced taste and smell disorders. Incidence, mechanisms and management related primarily to treatment of sensory receptor dysfunction. Drug Saf. (1994) 11:318–77. 10.2165/00002018-199411050-00004 7873092

[99] Moran DT Jafek BW Eller PM Rowley JC 3rd. Ultrastructural histopathology of human olfactory dysfunction. Microsc Res Tech. (1992) 23:103–10. 10.1002/jemt.1070230202 1421550

[100] Ackerman BH Kasbekar N. Disturbances of taste and smell induced by drugs. Pharmacotherapy. (1997) 17:482–96. 10.1002/j.1875-9114.1997.tb03058.x 9165552

[101] Murphy C Schubert CR Cruickshanks KJ Klein BE Klein R Nondahl DM. Prevalence of olfactory impairment in older adults. JAMA. (2002) 288:2307–12. 10.1001/jama.288.18.2307 12425708

[102] Fusari A Molina JA. (Sense of smell, physiological ageing and neurodegenerative diseases: II. Ageing and neurodegenerative diseases). Rev Neurol. (2009) 49:363–9. 19774531

[103] Lafreniere D Mann N. Anosmia: loss of smell in the elderly. Otolaryngol Clin North Am. (2009) 42:123–31. 10.1016/j.otc.2008.09.001 19134495

[104] Mullol J Alobid I Marino-Sanchez F Quinto L de Haro J Bernal-Sprekelsen M. Furthering the understanding of olfaction, prevalence of loss of smell and risk factors: a population-based survey (OLFACAT study). BMJ Open. (2012) 2:e001256. 10.1136/bmjopen-2012-001256 23135536 PMC3533119

[105] Jafek BW Gordon AS Moran DT Eller PM. Congenital anosmia. Ear Nose Throat J. (1990) 69:331–7. 2379478

[106] Getchell TV Subhedar NK Shah DS Hackley G Partin JV Sen G. Chemokine regulation of macrophage recruitment into the olfactory epithelium following target ablation: involvement of macrophage inflammatory protein-1alpha and monocyte chemoattractant protein-1. J Neurosci Res. (2002) 70:784–93. 10.1002/jnr.10432 12444600

[107] Barresi M Ciurleo R Giacoppo S Foti Cuzzola V Celi D Bramanti P. Evaluation of olfactory dysfunction in neurodegenerative diseases. J Neurol Sci. (2012) 323:16–24. 10.1016/j.jns.2012.08.028 23010543

[108] Yousem DM Geckle RJ Bilker WB Doty RL. Olfactory bulb and tract and temporal lobe volumes. Normative data across decades. Ann N Y Acad Sci. (1998) 855:546–55. 10.1111/j.1749-6632.1998.tb10624.x 9929650

[109] Doty RL. Olfaction in Parkinson's disease and related disorders. Neurobiol Dis. (2012) 46:527–52. 10.1016/j.nbd.2011.10.026 22192366 PMC3429117

[110] Woodward MR Amrutkar CV Shah HC Benedict RH Rajakrishnan S Doody RS. Validation of olfactory deficit as a biomarker of Alzheimer disease. Neurol Clin Pract. (2017) 7:5–14. 10.1212/CPJ.0000000000000293 28243501 PMC5310210

[111] Marin C Vilas D Langdon C Alobid I Lopez-Chacon M Haehner A. Olfactory dysfunction in neurodegenerative diseases. Curr Allergy Asthma Rep. (2018) 18:42. 10.1007/s11882-018-0796-4 29904888

[112] Berendse HW Ponsen MM. Diagnosing premotor Parkinson's disease using a two-step approach combining olfactory testing and DAT SPECT imaging. Parkinsonism Relat Disord. (2009) 15(Suppl. 3):S26–30. 10.1016/S1353-8020(09)70774-6 20083001

[113] Growdon ME Schultz AP Dagley AS Amariglio RE Hedden T Rentz DM. Odor identification and Alzheimer disease biomarkers in clinically normal elderly. Neurology. (2015) 84:2153–60. 10.1212/WNL.0000000000001614 25934852 PMC4451046

[114] Alves J Petrosyan A Magalhaes R. Olfactory dysfunction in dementia. World J Clin Cases. (2014) 2:661–7. 10.12998/wjcc.v2.i11.661 25405189 PMC4233420

[115] Doty RL Stern MB Pfeiffer C Gollomp SM Hurtig HI. Bilateral olfactory dysfunction in early stage treated and untreated idiopathic Parkinson's disease. J Neurol Neurosurg Psychiatry. (1992) 55:138–42. 10.1136/jnnp.55.2.138 1538221 PMC488979

[116] Shu CH Hummel T Lee PL Chiu CH Lin SH Yuan BC The proportion of self-rated olfactory dysfunction does not change across the life span. Am J Rhinol Allergy. (2009) 23:413–6. 10.2500/ajra.2009.23.3343 19671258

[117] Leonhardt B Tahmasebi R Jagsch R Pirker W Lehrner J. Awareness of olfactory dysfunction in Parkinson's disease. Neuropsychology. (2019) 33:633–41. 10.1037/neu0000544 30945913

[118] Mazal PP Haehner A Hummel T. Relation of the volume of the olfactory bulb to psychophysical measures of olfactory function. Eur Arch Otorhinolaryngol. (2016) 273:1–7. 10.1007/s00405-014-3325-7 25308243

[119] Pignatelli A Belluzzi O. Dopaminergic neurones in the main olfactory bulb: an overview from an electrophysiological perspective. Front Neuroanat. (2017) 11:7. 10.3389/fnana.2017.00007 28261065 PMC5306133

[120] Halasz N Nowycky M Hokfelt T Shepherd GM Markey K Goldstein M. Dopaminergic periglomerular cells in the turtle olfactory bulb. Brain Res Bull. (1982) 9:383–9. 10.1016/0361-9230(82)90149-6 6129042

[121] Yamaguchi M. Functional sub-circuits of the olfactory system viewed from the olfactory bulb and the olfactory tubercle. Front Neuroanat. (2017) 11:33. 10.3389/fnana.2017.00033 28443001 PMC5387040

[122] Escanilla O Yuhas C Marzan D Linster C. Dopaminergic modulation of olfactory bulb processing affects odor discrimination learning in rats. Behav Neurosci. (2009) 123:828–33. 10.1037/a0015855 19634942 PMC2766664

[123] Bonzano S Bovetti S Gendusa C Peretto P De Marchis S. Adult born olfactory bulb dopaminergic interneurons: molecular determinants and experience-dependent plasticity. Front Neurosci. (2016) 10:189. 10.3389/fnins.2016.00189 27199651 PMC4858532

[124] Bovetti S Veyrac A Peretto P Fasolo A De Marchis S. Olfactory enrichment influences adult neurogenesis modulating GAD67 and plasticity-related molecules expression in newborn cells of the olfactory bulb. PLoS ONE. (2009) 4:e6359. 10.1371/journal.pone.0006359 19626121 PMC2709916

[125] Huisman E Uylings HB Hoogland PV. A 100% increase of dopaminergic cells in the olfactory bulb may explain hyposmia in Parkinson's disease. Mov Disord. (2004) 19:687–92. 10.1002/mds.10713 15197709

[126] Sengoku R Matsushima S Bono K Sakuta K Yamazaki M Miyagawa S. Olfactory function combined with morphology distinguishes Parkinson's disease. Parkinsonism Relat Disord. (2015) 21:771–7. 10.1016/j.parkreldis.2015.05.001 25986741

[127] Paschen L Schmidt N Wolff S Cnyrim C van Eimeren T Zeuner KE. The olfactory bulb volume in patients with idiopathic Parkinson's disease. Eur J Neurol. (2015) 22:1068–73. 10.1111/ene.12709 25912367

[128] Scherfler C Schocke MF Seppi K Esterhammer R Brenneis C Jaschke W. Voxel-wise analysis of diffusion weighted imaging reveals disruption of the olfactory tract in Parkinson's disease. Brain. (2006) 129(Pt 2):538–42. 10.1093/brain/awh674 16272163

[129] Iannilli E Stephan L Hummel T Reichmann H Haehner A. Olfactory impairment in Parkinson's disease is a consequence of central nervous system decline. J Neurol. (2017) 264:1236–46. 10.1007/s00415-017-8521-0 28550478

[130] Su M Wang S Fang W Zhu Y Li R Sheng K. Alterations in the limbic/paralimbic cortices of Parkinson's disease patients with hyposmia under resting-state functional MRI by regional homogeneity and functional connectivity analysis. Parkinsonism Relat Disord. (2015) 21:698–703. 10.1016/j.parkreldis.2015.04.006 25937615

[131] Hubbard PS Esiri MM Reading M McShane R Nagy Z. Alpha-synuclein pathology in the olfactory pathways of dementia patients. J Anat. (2007) 211:117–24. 10.1111/j.1469-7580.2007.00748.x 17553102 PMC2375794

[132] Rey NL Wesson DW Brundin P. The olfactory bulb as the entry site for prion-like propagation in neurodegenerative diseases. Neurobiol Dis. (2018) 109(Pt B):226–l48. 10.1016/j.nbd.2016.12.013 28011307 PMC5972535

[133] Rey NL Steiner JA Maroof N Luk KC Madaj Z Trojanowski JQ. Widespread transneuronal propagation of alpha-synucleinopathy triggered in olfactory bulb mimics prodromal Parkinson's disease. J Exp Med. (2016) 213:1759–78. 10.1084/jem.20160368 27503075 PMC4995088

[134] Braak H Del Tredici K Rub U de Vos RA Jansen Steur EN Braak E. Staging of brain pathology related to sporadic Parkinson's disease. Neurobiol Aging. (2003) 24:197–211. 10.1016/S0197-4580(02)00065-9 12498954

[135] Haehner A Schopf V Loureiro A Linn J Reichmann H Hummel T. Substantia nigra fractional anisotropy changes confirm the PD at-risk status of patients with idiopathic smell loss. Parkinsonism Relat Disord. (2018) 50:113–6. 10.1016/j.parkreldis.2018.02.026 29477459

[136] Harding AJ Stimson E Henderson JM Halliday GM. Clinical correlates of selective pathology in the amygdala of patients with Parkinson's disease. Brain. (2002) 125(Pt 11):2431–45. 10.1093/brain/awf251 12390970

[137] Tsuboi Y Wszolek ZK Graff-Radford NR Cookson N Dickson DW. Tau pathology in the olfactory bulb correlates with Braak stage, lewy body pathology and apolipoprotein epsilon4. Neuropathol Appl Neurobiol. (2003) 29:503–10. 10.1046/j.1365-2990.2003.00453.x 14507342

[138] Fullard ME Morley JF Duda JE. Olfactory dysfunction as an early biomarker in parkinson's disease. Neurosci Bull. (2017) 33:515–25. 10.1007/s12264-017-0170-x 28831680 PMC5636737

[139] Cavaco S Goncalves A Mendes A Vila-Cha N Moreira I Fernandes J. Abnormal olfaction in parkinson's disease is related to faster disease progression. Behav Neurol. (2015) 2015:976589. 10.1155/2015/976589 26136625 PMC4468273

[140] Ponsen MM Stoffers D Wolters E Booij J Berendse HW. Olfactory testing combined with dopamine transporter imaging as a method to detect prodromal Parkinson's disease. J Neurol Neurosurg Psychiatry. (2010) 81:396–9. 10.1136/jnnp.2009.183715 19965851

[141] Fullard ME Tran B Xie SX Toledo JB Scordia C Linder C. Olfactory impairment predicts cognitive decline in early Parkinson's disease. Parkinsonism Relat Disord. (2016) 25:45–51. 10.1016/j.parkreldis.2016.02.013 26923521 PMC4825674

[142] Baba T Kikuchi A Hirayama K Nishio Y Hosokai Y Kanno S. Severe olfactory dysfunction is a prodromal symptom of dementia associated with Parkinson's disease: a 3 year longitudinal study. Brain. (2012) 135(Pt 1):161–9. 10.1093/brain/awr321 22287381

[143] Pardini M Huey ED Cavanagh AL Grafman J. Olfactory function in corticobasal syndrome and frontotemporal dementia. Arch Neurol. (2009) 66:92–6. 10.1001/archneurol.2008.521 19139305 PMC2987736

[144] Tijero B Gomez-Esteban JC Llorens V Lezcano E Gonzalez-Fernandez MC de Pancorbo MM. Cardiac sympathetic denervation precedes nigrostriatal loss in the E46K mutation of the alpha-synuclein gene (SNCA). Clin Auton Res. (2010) 20:267–9. 10.1007/s10286-010-0068-4 20443127

[145] Papadimitriou D Antonelou R Miligkos M Maniati M Papagiannakis N Bostantjopoulou S. Motor and nonmotor features of carriers of the p.A53T alpha-synuclein mutation: a longitudinal study. Mov Disord. (2016) 31:1226–30. 10.1002/mds.26615 27028329

[146] Malek N Swallow DM Grosset KA Lawton MA Smith CR Bajaj NP. Olfaction in parkin single and compound heterozygotes in a cohort of young onset Parkinson's disease patients. Acta Neurol Scand. (2016) 134:271–6. 10.1111/ane.12538 26626018

[147] Ferraris A Ialongo T Passali GC Pellecchia MT Brusa L Laruffa M. Olfactory dysfunction in parkinsonism caused by PINK1 mutations. Mov Disord. (2009) 24:2350–7. 10.1002/mds.22816 19890973

[148] Saunders-Pullman R Stanley K Wang C San Luciano M Shanker V Hunt A. Olfactory dysfunction in LRRK2 G2019S mutation carriers. Neurology. (2011) 77:319–24. 10.1212/WNL.0b013e318227041c 21753159 PMC3140803

[149] Healy DG Falchi M O'Sullivan SS Bonifati V Durr A Bressman S Phenotype, genotype, and worldwide genetic penetrance of LRRK2-associated Parkinson's disease: a case-control study. Lancet Neurol. (2008) 7:583–90. 10.1016/S1474-4422(08)70117-0 18539534 PMC2832754

[150] Baron JA Cigarette smoking and Parkinson's disease. Neurology. (1986) 36:1490–6. 10.1212/WNL.36.11.1490 3531917

[151] Gorell JM Rybicki BA Johnson CC Peterson EL. Smoking and Parkinson's disease: a dose-response relationship. Neurology. (1999) 52:115–9. 10.1212/WNL.52.1.115 9921857

[152] Lucassen EB Sterling NW Lee EY Chen H Lewis MM Kong L. History of smoking and olfaction in Parkinson's disease. Mov Disord. (2014) 29:1069–74. 10.1002/mds.25912 24833119 PMC4107167

[153] Vennemann MM Hummel T Berger K. The association between smoking and smell and taste impairment in the general population. J Neurol. (2008) 255:1121–6. 10.1007/s00415-008-0807-9 18677645

[154] Ajmani GS Suh HH Wroblewski KE Pinto JM. Smoking and olfactory dysfunction: a systematic literature review and meta-analysis. Laryngoscope. (2017) 127:1753–61. 10.1002/lary.26558 28561327 PMC6731037

[155] Breen DP Low HL Misbahuddin A. The impact of deep brain stimulation on sleep and olfactory function in Parkinson's disease. Open Neurol J. (2015) 9:70–2. 10.2174/1874205X01509010070 26535069 PMC4627383

[156] Silva MME Mercer PBS Witt MCZ Pessoa RR. Olfactory dysfunction in Alzheimer's disease systematic review and meta-analysis. Dement Neuropsychol. (2018) 12:123–32. 10.1590/1980-57642018dn12-020004 29988355 PMC6022986

[157] Kovacs T Cairns NJ Lantos PL. β-amyloid deposition and neurofibrillary tangle formation in the olfactory bulb in ageing and Alzheimer's disease. Neuropathol Appl Neurobiol. (1999) 25:481–91. 10.1046/j.1365-2990.1999.00208.x 10632898

[158] Wilson DA Xu W Sadrian B Courtiol E Cohen Y Barnes DC. Cortical odor processing in health and disease. Prog Brain Res. (2014) 208:275–305. 10.1016/B978-0-444-63350-7.00011-5 24767487 PMC4284974

[159] Franks KH Chuah MI King AE Vickers JC. Connectivity of pathology: the olfactory system as a model for network-driven mechanisms of Alzheimer's disease pathogenesis. Front Aging Neurosci. (2015) 7:234. 10.3389/fnagi.2015.00234 26696886 PMC4678206

[160] Wang J Eslinger PJ Doty RL Zimmerman EK Grunfeld R Sun X. Olfactory deficit detected by fMRI in early Alzheimer's disease. Brain Res. (2010) 1357:184–94. 10.1016/j.brainres.2010.08.018 20709038 PMC3515873

[161] Wesson DW Levy E Nixon RA Wilson DA. Olfactory dysfunction correlates with amyloid-beta burden in an Alzheimer's disease mouse model. J Neurosci. (2010) 30:505–14. 10.1523/JNEUROSCI.4622-09.2010 20071513 PMC2826174

[162] Yao ZG Hua F Zhang HZ Li YY Qin YJ. Olfactory dysfunction in the APP/PS1 transgenic mouse model of Alzheimer's disease: morphological evaluations from the nose to the brain. Neuropathology. (2017) 37:485–94. 10.1111/neup.12391 28643854

[163] Larson J Hoffman JS Guidotti A Costa E. Olfactory discrimination learning deficit in heterozygous reeler mice. Brain Res. (2003) 971:40–6. 10.1016/S0006-8993(03)02353-9 12691835

[164] Brai E Marathe S Zentilin L Giacca M Nimpf J Kretz R. Notch1 activity in the olfactory bulb is odour-dependent and contributes to olfactory behaviour. Eur J Neurosci. (2014) 40:3436–49. 10.1111/ejn.12719 25234246

[165] Alvarado-Martinez R Salgado-Puga K Pena-Ortega F. Amyloid beta inhibits olfactory bulb activity and the ability to smell. PLoS ONE. (2013) 8:e75745. 10.1371/journal.pone.0075745 24086624 PMC3784413

[166] Bahar-Fuchs A Chetelat G Villemagne VL Moss S Pike K Masters CL. Olfactory deficits and amyloid-beta burden in Alzheimer's disease, mild cognitive impairment, and healthy aging: a PiB PET study. J Alzheimers Dis. (2010) 22:1081–7. 10.3233/JAD-2010-100696 20930316

[167] Sun GH Raji CA Maceachern MP Burke JF. Olfactory identification testing as a predictor of the development of Alzheimer's dementia: a systematic review. Laryngoscope. (2012) 122:1455–62. 10.1002/lary.23365 22552846

[168] Risacher SL Tallman EF West JD Yoder KK Hutchins GD Fletcher JW Olfactory identification in subjective cognitive decline and mild cognitive impairment: association with tau but not amyloid positron emission tomography. Alzheimers Dement (Amst). (2017) 9:57–66. 10.1016/j.dadm.2017.09.001 29159268 PMC5675709

[169] Mufson EJ Counts SE Perez SE Ginsberg SD. Cholinergic system during the progression of Alzheimer's disease: therapeutic implications. Expert Rev Neurother. (2008) 8:1703–18. 10.1586/14737175.8.11.1703 18986241 PMC2631573

[170] Pelton GH Soleimani L Roose SP Tabert MH Devanand DP. Olfactory deficits predict cognitive improvement on donepezil in patients with depression and cognitive impairment: a randomized controlled pilot study. Alzheimer Dis Assoc Disord. (2016) 30:67–9. 10.1097/WAD.0000000000000107 26398910 PMC4764438

[171] Braak H Braak E. Neuropathological stageing of Alzheimer-related changes. Acta Neuropathol. (1991) 82:239–59. 10.1007/BF00308809 1759558

[172] Moberg PJ Doty RL. Olfactory function in Huntington's disease patients and at-risk offspring. Int J Neurosci. (1997) 89:133–9. 10.3109/00207459708988468 9134451

[173] Larsson M Lundin A Robins Wahlin TB. Olfactory functions in asymptomatic carriers of the Huntington disease mutation. J Clin Exp Neuropsychol. (2006) 28:1373–80. 10.1080/13803390500473746 17050264

[174] Gunther R Richter N Sauerbier A Chaudhuri KR Martinez-Martin P Storch A. Non-motor symptoms in patients suffering from motor neuron diseases. Front Neurol. (2016) 7:117. 10.3389/fneur.2016.00117 27504105 PMC4958907

[175] Viguera C Wang J Mosmiller E Cerezo A Maragakis NJ. Olfactory dysfunction in amyotrophic lateral sclerosis. Ann Clin Transl Neurol. (2018) 5:976–81. 10.1002/acn3.594 30128322 PMC6093848

[176] Pilotto A Rossi F Rinaldi F Compostella S Cosseddu M Borroni B. Exploring olfactory function and its relation with behavioral and cognitive impairment in amyotrophic lateral sclerosis patients: a cross-sectional study. Neurodegener Dis. (2016) 16:411–6. 10.1159/000446802 27497596

[177] Gunther R Schrempf W Hahner A Hummel T Wolz M Storch A. Impairment in respiratory function contributes to olfactory impairment in amyotrophic lateral sclerosis. Front Neurol. (2018) 9:79. 10.3389/fneur.2018.00079 29535673 PMC5834512

[178] Takeda T Iijima M Uchihara T Ohashi T Seilhean D Duyckaerts C. TDP-43 pathology progression along the olfactory pathway as a possible substrate for olfactory impairment in amyotrophic lateral sclerosis. J Neuropathol Exp Neurol. (2015) 74:547–56. 10.1097/NEN.0000000000000198 25933387

[179] Takeda T Uchihara T Kawamura S Ohashi T. Olfactory dysfunction related to TDP-43 pathology in amyotrophic lateral sclerosis. Clin Neuropathol. (2014) 33:65–7. 10.5414/NP300661 24131749

[180] Scangas GA Bleier BS. Anosmia: differential diagnosis, evaluation, and management. Am J Rhinol Allergy. (2017) 31:3–7. 10.2500/ajra.2017.31.4403 28234141

[181] Gudziol V Hummel T. Effects of pentoxifylline on olfactory sensitivity: a postmarketing surveillance study. Arch Otolaryngol Head Neck Surg. (2009) 135:291–5. 10.1001/archoto.2008.524 19289709

[182] Pekala K Chandra RK Turner JH. Efficacy of olfactory training in patients with olfactory loss: a systematic review and meta-analysis. Int Forum Allergy Rhinol. (2016) 6:299–307. 10.1002/alr.21669 26624966 PMC4783272

[183] Kattar N Do TM Unis GD Migneron MR Thomas AJ McCoul ED. Olfactory training for postviral olfactory dysfunction: systematic review and meta-analysis. Otolaryngol Head Neck Surg. (2020). 10.1177/0194599820943550. [Epub ahead of print]. 32660334

[184] Reichert JL Schopf V. Olfactory loss and regain: lessons for neuroplasticity. Neuroscientist. (2018) 24:2–35. 10.1177/1073858417703910 28459173

